# Sparse-firing regularization methods for spiking neural networks with time-to-first-spike coding

**DOI:** 10.1038/s41598-023-50201-5

**Published:** 2023-12-21

**Authors:** Yusuke Sakemi, Kakei Yamamoto, Takeo Hosomi, Kazuyuki Aihara

**Affiliations:** 1 Research Center for Mathematical Engineering, Chiba Institute of Technology, Narashino, Japan; 2 International Research Center for Neurointelligence (WPI-IRCN), The University of Tokyo, Tokyo, Japan; 3 Massachusetts Institute of Technology, Cambridge, USA; 4 NEC Corporation, Kawasaki, Japan

**Keywords:** Computer science, Applied mathematics

## Abstract

The training of multilayer spiking neural networks (SNNs) using the error backpropagation algorithm has made significant progress in recent years. Among the various training schemes, the error backpropagation method that directly uses the firing time of neurons has attracted considerable attention because it can realize ideal temporal coding. This method uses time-to-first-spike (TTFS) coding, in which each neuron fires at most once, and this restriction on the number of firings enables information to be processed at a very low firing frequency. This low firing frequency increases the energy efficiency of information processing in SNNs. However, only an upper limit has been provided for TTFS-coded SNNs, and the information-processing capability of SNNs at lower firing frequencies has not been fully investigated. In this paper, we propose two spike-timing-based sparse-firing (SSR) regularization methods to further reduce the firing frequency of TTFS-coded SNNs. Both methods are characterized by the fact that they only require information about the firing timing and associated weights. The effects of these regularization methods were investigated on the MNIST, Fashion-MNIST, and CIFAR-10 datasets using multilayer perceptron networks and convolutional neural network structures.

## Introduction

Spiking neural networks (SNNs) can process information in the form of spikes in a manner similar to the way information is processed in the brain. SNNs are thereby expected to be able to achieve both high computational functionality and energy efficiency^1^. The spikes are represented as all-or-none binary values, and how information is represented by spikes is closely related to the information-processing mechanism in SNNs. The spike-based information representation methods are divided into two major categories, rate coding and temporal coding^2,3^. In rate coding, information is contained in the average number of spikes generated by a neuron. In this case, the firing frequency can take approximately continuous values as a function of the input intensities; therefore, the resulting SNNs can be treated as differentiable models similar to an artificial neural network (ANN). Using rate coding, ANNs can be converted to SNNs, and the high learning ability of ANNs has been successfully transferred to SNNs^4–6^. However, when rate coding is used, information processing in the SNNs is just an approximation of that in ANNs. Furthermore, the precise approximation of an ANN requires many spikes, which reduces energy efficiency when implemented in neuromorphic hardware^7^. It has been experimentally shown that physiologically, neurons in certain brain regions or specific neuron types exhibit extremely sparse firing characteristics^8^, and it is thought that temporal coding using not only the firing frequency but also the firing time is realized in at least some brain regions^9–13^. Therefore, to achieve brain-like high-capacity, energy-efficient information processing capabilities in SNNs, it is important to use temporal coding that also considers spike timing information.

Because in temporal coding, the precise timing of spikes carries information, it is necessary to train the SNNs directly instead of using a converted ANN. In recent years, by incorporating deep learning techniques, it has become possible to directly train SNNs using the backpropagation algorithm^14–17^. Among the various methods proposed, methods that focus on the displacement of the membrane potential and those that focus on the displacement of the spike time have attracted particular attention because of their high learning performance. In membrane potential displacement methods, the derivative of the output spike with respect to the membrane potential is almost always zero because the spike is a binary value. However, it is possible to approximate this derivative using a surrogate function^18^. This method has been proposed in various forms by various groups^19–22^. It has been proven to be very flexible, works with various surrogate functions^23^, and can be used to efficiently train recurrent neural network structures^24,25^. Recently, it has become possible to train relatively large models^26^. However, with few exceptions^27–29^, the neurons exhibit high firing frequencies, and it is debatable whether the timing information is efficiently utilized.

A timing-based learning method is a method that focuses on the displacement of the spike time^30^. The coding most commonly used in this learning method is time-to-first-spike (TTFS) coding, which has the property that each neuron fires at most once^31,32^. Because the information is contained in the timing of a single spike and the gradient is computed directly using the spike timing, this coding is expected to realize an ideal temporal coding. The high learning performance of this method has been shown in various neuron models^33–39^. Hardware implementation efforts are also underway to achieve high power efficiency by taking advantage of its sparsity characteristics^39,40^. However, the constraint of firing at most once per neuron in TTFS coding may not be sufficiently sparse in some situations. For example, in the brain, there are many neurons that hardly fire at all^8^. Furthermore, in an extremely power-limited environment such as edge AI^41^, a sparse firing pattern that goes beyond the constraint of one firing per neuron is desirable.

In this paper, we propose two methods to further improve the sparse firing property of TTFS-coded SNNs. One method is derived from the loss in the value of the membrane potential, and the other is derived from the firing conditions. Both methods are characterized by the fact that they only require information about the firing timing and the weights associated with it, as is the case in timing-based learning. In the following, we describe the two methods and show experimentally how they suppress firing effectively on the MNIST, Fashion-MNIST, and CIFAR-10 datasets.

## Results

### Spike-timing-based sparse-firing regularization methods

**Figure 1:**
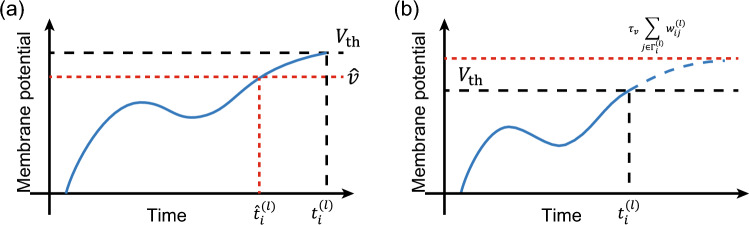
Derivation of the two SSR methods. (**a**) M-SSR is derived from the membrane potential loss, where the loss occurs when the membrane potential is larger than ${\hat{v}}$. Assuming that a neuron fires at $t_i^{(l)}$ and ${\hat{v}}$ is sufficiently large, the loss occurs only in the time interval $[{\hat{t}}_i^{(l)}, t_i^{(l)}]$, where ${\hat{t}}_i^{(l)}$ is the time at which the membrane potential equals ${\hat{v}}$. M-SSR is obtained by setting ${\hat{v}}\rightarrow V_\text {th}$. (**b**) F-SSR is derived from the firing condition. Assuming that no input spike is accepted after the neuron’s firing time $t_i^{(l)}$, the membrane potential asymptotes to $\tau _v \sum _{j\in \Gamma _i^{(l)}}w_{ij}^{(l)}$ at $t\rightarrow \infty$. The F-SSR is derived by formulating the loss such that this asymptotic membrane potential becomes small.

We first summarize the proposed spike-timing-based sparse-firing regularization (SSR) methods. SSR methods are characterized by the fact that they only require information about the firing timing and the weights associated with it, as is the case in ordinary timing-based learning. In this study, we propose two SSR method variants: membrane-potential-aware SSR (M-SSR) and firing-condition-aware SSR (F-SSR). In both cases, we add a new regularization term to the cost function used in supervised learning to suppress the firing. M-SSR is based on the idea of reducing the value of the membrane potential, which is realized by adding the membrane potential loss *V* as a regularization term to the cost function. F-SSR is based on the idea of breaking the firing conditions, which is realized by adding the firing condition loss *Q* as a regularization term to the cost function. Figure 1 shows the outline of each method. For simplicity, this paper adopts commonly used leaky integrate-and-fire (LIF) neuron models. The LIF neuron model has as parameters the time constant of the membrane potential $\tau _v$ and the time constant of the synaptic current $\tau _I$ (see “Method”). Extensions to other neuron models are straightforward.

First, we explain M-SSR, which is based on the idea of reducing the membrane potential value. The membrane potential loss *V* is defined as

1 $$\begin{aligned} V&= \sum _l \xi ^l \sum _i V_i^{(l)}, \end{aligned}$$

2 $$\begin{aligned} V_i^{(l)}&= \frac{1}{V_\text {th}-{\hat{v}}}\int _0 ^T dt \left( v_i^{(l)}(t) - {\hat{v}} \right) \theta \left( v_i^{(l)}(t) - {\hat{v}} \right) \theta \left( t_i^{(l)} - t \right) , \end{aligned}$$

where $V_i^{(l)}$ is the loss relating to the membrane potential trajectory of the *l*th-layer neuron *i* and $\xi (>0)$ is the hyperparameter for leveling the sparsity in each layer. In addition, $V_\text {th}$ is the firing threshold, and *T* is a parameter specifying the time interval during which firing is suppressed. Figure 1 (a) shows the loss associated with the membrane potential trajectory of a neuron. Note that ${\hat{v}}$ is sufficiently large and there is only one point ${\hat{t}}_i^{(l)}$ at which the membrane potential equals ${\hat{v}}$. In this case, the loss is nonzero only during $[{\hat{t}}_i^{(l)}, t_i^{(l)}]$. We note that the regularization with Eq. (2) can be regarded as previously proposed methods^27–29^, as explained in the “Discussion” section. To perform integration in Eq. (2), we need information about the value of the membrane potential at each time step. However, by setting ${\hat{v}}\rightarrow V_\text {th}$, we can obtain ${\hat{t}}_i^{(l)} \rightarrow t_i^{( l)}$. This avoids the integral calculation, and Eq. (2) can be solved analytically. When computing the gradient of this integral, it is important to fix the integration range $[{\hat{t}}, t_i^{(l)}]$. If we do not treat it as a fixed value, the more rapidly the membrane potential rises, the smaller the membrane potential loss in Eq. (2) becomes, and thus the firing is not effectively suppressed. Finally, we obtain the following M-SSR:3Note that in the above equations, the gradients are not calculated for the variables shown in blue (they are considered to be constants in the gradient calculations). This corresponds to fixing the integration range $[{\hat{t}}, t_i^{(l)}]$. Constant terms not involved in the learning are excluded. $\Gamma _i^{(l)}$ denotes the index set of spikes that have been input to the *l*th-layer neuron *i* up to firing time $t_i^{(l)}$. In Eq. (3), the following variables are defined

4 $$\begin{aligned} a_i^{(l)}&= \sum _{j\in \Gamma _i^{(l)}} w_{ij}^{(l)} \exp \left( \frac{t_j^{(l-1)}}{\tau } \right) ,~ b_i^{(l)}= \sum _{j\in \Gamma _i^{(l)}} w_{ij}^{(l)} \exp \left( \frac{t_j^{(l-1)}}{2\tau } \right) , \end{aligned}$$

5 $$\begin{aligned} \alpha _i^{(l)}&=\frac{2a_i^{(l)}}{\left( b_i^{(l)} + \sqrt{\left( b_i^{(l)}\right) ^2-2a_i^{(l)}\tau ^{-1}V_\text {th}}\right) \left( \sqrt{\left( b_i^{(l)}\right) ^2 - 2a_i^{(l)}\tau ^{-1}V_\text {th}}\right) }. \end{aligned}$$

See the Supplementary Material for a detailed derivation. In addition, the Supplementary Material discusses the consistency of the M-SSR gradient (Eq. 3) with that of the integral-form loss (Eq. 2) when ${\hat{v}}\rightarrow V_\text {th}$ (see Supplementary Fig. 1).

Next, we explain F-SSR, a method that suppresses firing based on the firing conditions. For the case of the non-leaky integrate-and-fire neuron model $(\tau _v = \infty ,~\tau _I = \tau )$, we obtain the following firing conditions:

6 $$\begin{aligned} \text {firing condition}_i^{(l)} := \sum _{j\in \Gamma _i^{(l)}} w_{ij}^{(l)} \ge V_\text {th} \tau ^{-1}. \end{aligned}$$

Because the firing will be suppressed if this firing condition is not satisfied, we define the F-SSR term *Q* as follows:

7 $$\begin{aligned} Q&= \sum _{l} \xi ^l \sum _i Q_i^{(l)} \end{aligned}$$

8 $$\begin{aligned} Q_i^{(l)}&= {\left\{ \begin{array}{ll} \sum _{j\in \Gamma _i^{(l)}} w_{ij}^{(l)}, ~\text {if } t_i^{(l)} < T \\ 0,~\text {otherwise.} \\ \end{array}\right. } \end{aligned}$$

We note that $V_i^{(l)}=Q_i^{(l)}=0$ if the neuron does not fire.

### Numerical simulations

We trained several SNNs on the MNIST dataset^42^, Fashion-MNIST dataset^43^, and CIFAR-10 dataset^44^ to investigate the effect of SSR on suppressing firing. In the experiment, in addition to the multilayer perceptron (MLP) structure, a convolutional neural network (CNN) structure was used. The image data in the dataset were converted to input spikes, where the intensity of each pixel is converted to the input time of each spike (see Method). We define sparsity as the average number of spikes per neuron per input data in a time window $[0,t^\text {ref}]$, where $t^\text {ref}$ is the reference time of the output layer firing time (see “Method”). In addition, we set $T=t^\text {ref}$ in Eqs. (2) and (8), and set the firing threshold $V_\text {th}$ to 1. When the integration form Eq. (2) was used, the integration was approximated by dividing the integral by the time width $\Delta t$.

**Figure 2:**
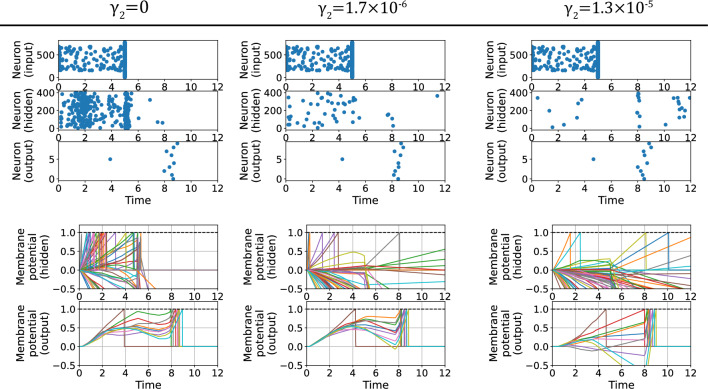
Typical results with M-SSR. We trained SNNs with one hidden layer (784-400-10) on the MNIST dataset with various values of M-SSR strengths $\gamma _2$. The upper figures present raster plots for a given input data, showing the results of the input layer (top), hidden layer (middle), and output layer (bottom). The lower figures show the time evolution of the membrane potentials when given the same input data, with the results for the hidden layer (top) and the output layer (bottom). In the panels displaying the membrane potentials in the hidden layer, only 50 neurons are shown. In all cases, the following hyperparameters were used: $\tau _v = \tau _I = \infty ,~t^\text {ref}=8,~\gamma _1 = 10^{-4}, \gamma _3=0, \eta = 10^{-4}$, and $\tau _\text {soft}=0.9$.

Figure 2 shows the learning results of SNNs with one hidden layer (784-400-10) trained on the MNIST dataset with various M-SSR strengths $\gamma _2$ (see “Method”). Note that all output layer neurons were required to fire because the loss function was defined by the spike timing of the neurons in the output layer. Therefore, sparse firing regularization was applied only to the hidden layers. The upper figures show the raster plots of the firing distribution of each layer for a given input data, and the lower figures show the time evolution of the membrane potentials of each layer. As the strength of M-SSR was increased, the number of neurons that fired tended to decrease, and it can be seen that most of the hidden layer neurons stopped firing when $\gamma _2=1.3\times 10^{-5}$. By contrast, the firing distribution of the output layer did not change significantly with respect to M-SSR strength. The neurons corresponding to the correct index fired the earliest $(t\sim 4)$, and the other neurons fired later $(t\sim 8)$. The membrane potentials of the hidden layer neurons were suppressed as the regularization strength increased, whereas the output layer solved the task using fewer spikes from the hidden layer. This indicates that M-SSR regularization can suppress the firing of the hidden layer without significantly compromising recognition task performance.

**Figure 3:**
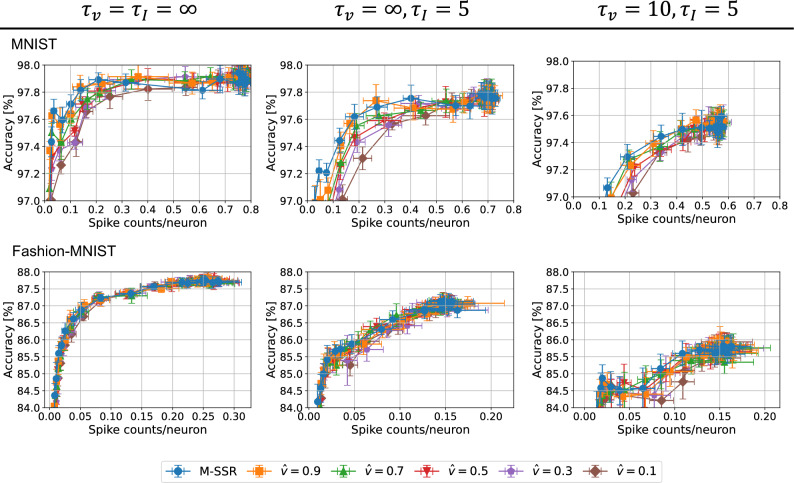
Sparsity–accuracy tradeoff for different regularization forms in 2-layer SNNs.. We evaluated the integral-form regularization (Eq. 2) and M-SSR (Eq. 3) in a 784-400-10 SNN based on various neuron models. The tradeoff in sparsity–accuracy is shown when the regularization strength $\gamma _2$ was varied from 0 to $10^{-4}$. The standard deviations were obtained over 10 trials. The hyperparameters were $t^\text {ref}=8, \gamma _1 = 10^{-4}, \gamma _3=0, \eta = 10^{-4}$, and $\tau _\text {soft}=0.9$. For the integral-form regularization, we used various values of ${\hat{v}}$, and we set $\Delta t = t^\text {ref}/1000$.

**Figure 4:**
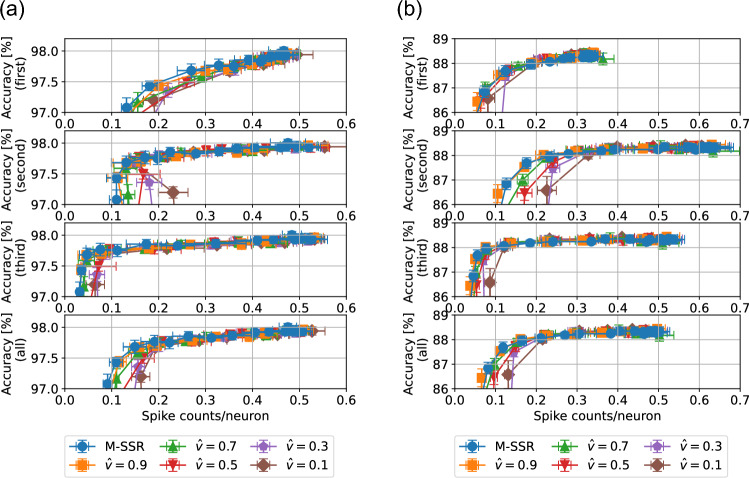
Sparsity–accuracy tradeoff for different regularization forms in 4-layer SNNs. We evaluated the integral-form regularization (Eq. 2) and M-SSR (Eq. 3) in a 784-400-400-400-10 SNN based on the neuron model of $\tau _v=\tau _I=\infty$ for the MNIST dataset (**a**) and Fashion-MNIST dataset (**b**). In (**a,b**), each panel represents the accuracy–sparsity tradeoff for the first, second, and third hidden layers, from the top. The bottom panel presents the sparsity–accuracy tradeoffs for the sparsity averaged over the three hidden layers. The tradeoff in sparsity–accuracy is shown when the regularization strength $\gamma _2$ was varied from 0 to $10^{-4}$. The standard deviations were obtained over 10 trials. The hyperparameters were $t^\text {ref}=9, \gamma _1 = 10^{-4}, \gamma _3=0, \eta = 10^{-4}$, $\tau _\text {soft}=0.9$, and $\xi =6$. For the integral-form regularization, we used various values of ${\hat{v}}$, and we set $\Delta t = t^\text {ref}/1000$.

Figure 3 shows the sparsity–accuracy tradeoff results obtained when using the integral-form regularization (Eq. 2) and M-SSR (Eq. 3). We trained SNNs with a single hidden layer (784-400-10) using various regularization strengths. The standard deviations were obtained over 10 trials. The upper figures show the results for the MNIST dataset, and the lower figures show the results for the Fashion-MNIST dataset. Results are also shown for different neuron models $(\tau _v, \tau _I)$. For the MNIST dataset, the tradeoff curves show that a larger ${\hat{v}}$ led to a better tradeoff for all neuron models, and the best tradeoff was obtained by M-SSR (corresponding to ${\hat{v}}=1$). Similar results were obtained for Fashion-MNIST, although the advantage was not as pronounced as it was for MNIST. This result demonstrates that the integral-form regularization (Eq. 2) smoothly transitioned to the limit form (M-SSR, Eq. 3). Furthermore, taking the limit of ${\hat{v}}\rightarrow 1$, the tradeoff between sparsity and accuracy was improved. In addition, good sparsity–accuracy tradeoff properties were obtained for various neuron models ($\tau _v$ and $\tau _I$). Similarly, in Fig. 4, we evaluated the sparsity–accuracy tradeoff for SNNs with three hidden layers (784-400-400-400-10) using the neuron model of $\tau _v=\tau _I=\infty$. The value of $\xi$ (Eq. 1) was set to achieve the best tradeoff averaged across the entire layer, excluding the output layer. See supplementary Fig. 2 for the tradeoff properties for various values of $\xi$. For the both cases of the MNIST dataset (Fig. 4a) and Fashion-MNIST dataset (Fig. 4b), we obtained the best tradeoff using M-SSR, followed by integral-form regularization. These results obtained in Figs. 3 and 4 suggest that M-SSR is preferable to the integral-form regularization for TTFS-coded SNNs. We note that the integral-form regularization can be regarded as previously proposed methods^27–29^, as explained in the “Discussion” section.

**Figure 5:**
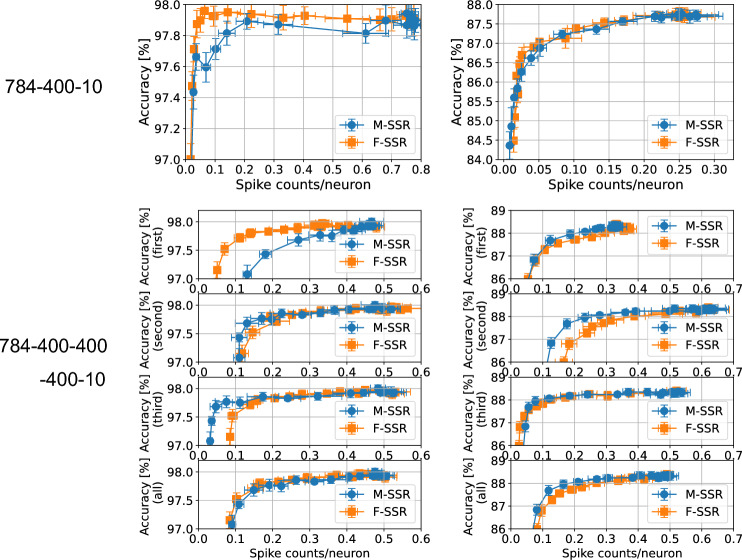
Comparison of SSR methods on the MNIST and Fashion-MNIST datasets. The top figures show the sparsity–accuracy tradeoffs for the 2-layer SNNs (784-400-10), whereas the bottom figures show the sparsity–accuracy tradeoffs for the 4-layer SNNs (784-400-400-400-10). In the bottom figures, each panel represents the accuracy–sparsity tradeoff for the first, second, and third hidden layers, from the top. The bottom panel presents the sparsity–accuracy tradeoffs for the sparsity averaged over the three hidden layers. The tradeoff in sparsity–accuracy is shown when the regularization strength $\gamma _2$ and $\gamma _3$ was varied from 0 to $10^{-4}$ for M-SSR, and from 0 to $10^{-3}$ for F-SSR, respectively. The standard deviations were obtained over 10 trials. We used the following hyperparameters: $t^\text {ref}=8, \gamma _1 = 10^{-4}, \eta = 10^{-4}$, and $\tau _\text {soft}=0.9$ for the 2-layer SNNs, $t^\text {ref}=9, \gamma _1 = 10^{-4}, \eta = 10^{-4}$, and $\tau _\text {soft}=0.9$ for the 4-layer SNNs. For the MNIST dataset, we set $\xi$ to 6 for M-SSR, and 1 for F-SSR. For the Fashion-MNIST dataset, we set $\xi$ to 6 for M-SSR, and 4 for F-SSR.

Figure 5 compares the results of the two proposed SSRs, M-SSR (Eq. 3) and F-SSR (Eq. 8). The results for an SNN with one hidden layer (784-400-10) are shown in the top figures. On the MNIST benchmark, F-SSR obtained a better sparsity–accuracy tradeoff than M-SSR, whereas on the Fashion-MNIST benchmark, both F-SSR and M-SSR yielded a similar sparsity–accuracy tradeoff. The results for an SNN with three hidden layers are shown in the lower figures. For each SSR method for each dataset, the value of $\xi$ (Eq. 1) was set to obtain the best tradeoff averaged over the whole layer (except for the output layer). See supplementary Fig. 2 for the tradeoff properties for various values of $\xi$. For an SNN with three hidden layers, M-SSR and F-SSR showed similar sparsity–accuracy tradeoff characteristics, but the optimal value of $\xi$ differed significantly in M-SSR and F-SSR. For the MNIST dataset, the optimal value was $\xi =6$ for M-SSR and $\xi =1$ for F-SSR, whereas for the Fashion-MNIST dataset, the optimal value was $\xi =6$ for M-SSR and $\xi =4$ for F-SSR. This difference may be due to the characteristics of the regularization function. In M-SSR (Eq. 3), the error that occurred in the *l*-th layer propagates back to the previous layer via spike timing $t_j^{(l-1)}$. In this case, if the weights of neurons from $t_j^{(l-1)}$ to the *l*th layer were positive overall, $t_j^{(l-1)}$ increased during training, and consequently the $l-1$th layer also became more sparse. Similarly, the $l-2$th layer was expected to become sparse. Therefore, to counteract this effect, a relatively large value of $\xi$ was optimal. By contrast, in the case of F-SSR (Eq. 8), the losses that occurred in the *l*th layer do not propagate back to previous layers. Therefore, a relatively small value of $\xi$ was optimal.

**Table 1:** Convolutional architectures used for each dataset.

Dataset	Network
MNIST	Conv(5, 6)-Pool-Conv(5, 16)-Pool-400-400-10
Fashion-MNIST	Conv(5, 6)-Pool-Conv(5, 16)-Pool-400-400-10
CIFAR-10	Conv(3, 32)-Pool-Conv(3, 64)-Pool-Conv(3, 128)-Pool-600-10

“Conv(*a*,*b*)” represents a convolutional layer with a kernel size of $a\times a$, number of output channels *b*, and stride 1. “Pool” represents a pooling layer with kernel size of $2\times 2$, stride 2. The padding of the convolutional layer was set to 0 for MNIST and set to 1 for Fashion-MNIST and CIFAR-10.

**Figure 6:**
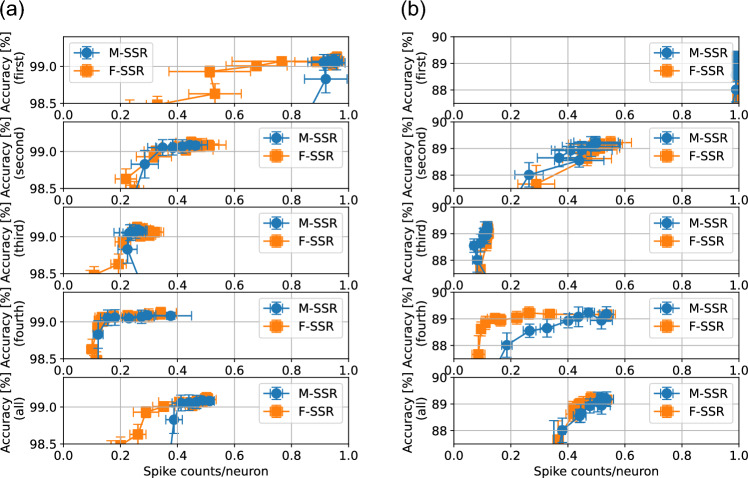
Effects of SSR with a CNN-architecture on the MNIST dataset (**a**) and the Fashion-MNIST dataset (**b**). From top to bottom, the panels represent the sparsity–accuracy tradeoffs for the first convolutional layer, second convolutional layer, first fully connected layer, second fully connected layer, and whole network (not including pooling and output layers). The tradeoff in sparsity–accuracy is shown when the regularization strength $\gamma _2$ and $\gamma _3$ was varied from 0 to $10^{-6}$ for M-SSR, and from 0 to $10^{-5}$ for F-SSR, respectively. The standard deviations were obtained over 10 trials. We used the following hyperparameters: $t^\text {ref}=16, \gamma _1 = 10^{-4}, \eta = 10^{-4},$ and $\tau _\text {soft}=0.9$. For the MNIST dataset, we set $\xi$ to 6 for M-SSR, and 2 for F-SSR. For the Fashion-MNIST dataset, we set $\xi$ to 6 for M-SSR, and 4 for F-SSR.

Next, we applied the SSR methods to spiking CNNs. Table 1 shows the network structure used for each dataset. Figure 6 shows the effect of SSR regularization on MNIST and Fashion-MNIST. The overall sparsity–accuracy tradeoff for the CNN structure was worse than that for the MLP structure. The SNNs with MLP structures reduced the average number of firings per neuron to about 0.1–0.2 with almost no loss in accuracy, whereas the SNNs with convolutional structures only reduced the number of firings to about 0.4. It can be seen that the first convolutional layers are not very sparse, with the exception of the results of F-SSR on the MNIST dataset. This is considered to be caused by the fact that it is difficult to force only a portion of the neurons to fire in a convolutional layer because of the weight-sharing property.

**Figure 7:**
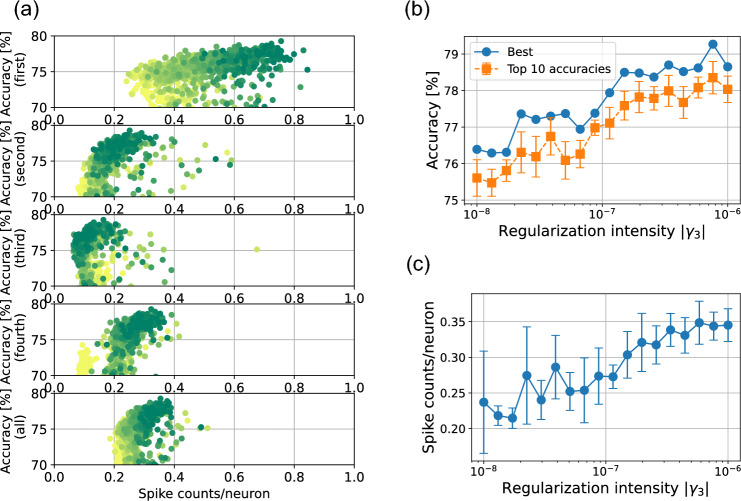
Sparsity–accuracy tradeoff on the CIFAR-10 task. Negative values of F-SSR $\gamma _3$ is used to promote the neurons to fire in order to stabilize the learning process. Nineteen values of $\gamma _3$ in the range of 0 to $-10^{-6}$ were evaluated. For each $\gamma _3$ value, we conducted 50 experiments with different weight initializations. (**a**) The sparsity-accuracy tradeoffs for the 950 experimental results. From the top, the sparsity–accuracy tradeoffs for the first, second, and third convolutional layers, and then fully connected layer are represented, respectively. The sparsity-accuracy tradeoffs for the whole network (not including pooling and output layers) is presented in the bottom panel. The color of the plots represents the value of $\gamma _3$: when $\gamma _3 = 0$, the color is yellow. As the value approaches $-10^{-6}$, the color gradually changes to green. (**b**) The best accuracy among 50 different weight initializations is ploted as a function of $\gamma _3$. Additionally, We present the mean and standard deviation of the top 10 accuracies. (**c**) The spike counts per neuron for the whole network (not including pooling and output layers) as a function of $\gamma _3$ are shown. The mean and standard deviation of spike counts are obtrained with 10 results that exhibit the highest accuracies.

On the CIFAR-10 dataset, firing tended to be suppressed during training even when SSR was not applied, and we confirmed that neurons relating to some channels did not fire at all image locations and for all training data. To avoid this problem, we trained the SNNs to attempt to satisfy the firing conditions. This was achieved by making the regularization strength $\gamma _3$ of the F-SSR term negative (see “Method”). The results of learning by promoting firing are shown in Fig.7a. Due to the large variance accross trials, we plotted the 50 results on the sparsity-accuracy map for each value of $\gamma _3$ to enhance visualization. By increasing the value of $|\gamma _3|$ (causing the color of plots to change from yellow to green), one can observe that the accuracy tends to increase while sacrificing sparsity. The best accuracy was 79.26% when $\gamma _3$ was $7.6\times 10^{-7}$. The performance improvement is clearly observed in Fig. 7b, illustrating the best and top 10 accuracies as a function of $\gamma _3$ are shown. Meanwhile, Fig. 7c depicts the change in sparsity as a function of $\gamma _3$.

## Discussion

SNNs with TTFS coding can realize ideal temporal coding by constraining each neuron to fire at most once. Due to this mechanism, the SNNs with TTFS coding have high firing sparsity, and this approach has been applied in energy-efficient hardware implementations^39,40^. To further improve this sparse firing characteristic, we developed the SSR methods. The two SSR methods were derived from two different perspectives. The first one is M-SSR, which was derived by assigning a penalty each time the membrane potential exceeds a threshold ${\hat{v}}$ and taking the limit when the threshold equals the firing threshold. The other is F-SSR, which was obtained from the firing conditions of neurons. Both SSR methods are characterized by the fact that they do not require information about the membrane potential itself, only the firing time and associated weights. The sparsity–accuracy properties of these two methods were investigated using the MNIST, Fashion-MNIST, and CIFAR-10 datasets. Interestingly, although some differences were observed depending on the datasets and the network structure, both F-SSR and M-SSR showed equally good sparsity–accuracy properties, even though the regularization methods were derived from different perspectives. In particular, for the fully connected layer, it was found that the average number of firings for each neuron could be lowered to 0.1 to 0.2. From the experiments conducted in this study, it is difficult to determine which method is superior. We can at least conclude that F-SSR has the advantage of a somewhat smaller computational load than M-SSR due to its simpler formula. To understand the difference between F-SSR and M-SSR, in addition to the sparsity–accuracy property, a detailed analysis of the changes in the firing characteristics and in the information processing mechanism associated with the sparse firing mechanism will be required in future.

For the CNN structures, we found the SSR methods had more difficulty suppressing the firing of neurons in the convolutional layer than in the fully connected layer. In CIFAR-10 in particular, we observed that firing is suppressed too much and learning becomes difficult even without SSR. This may be because it is difficult to flexibly decide whether the outputs belonging to a certain kernel should fire depending on the position because of the weight-sharing property. To prevent this, we found that, in CIFAR-10, learning performance can be improved by promoting firing. Similar firing promotion terms have been introduced in previous studies^33,45^. In timing-based learning of large-scale CNN structures, one way to obtain better sparsity–accuracy properties is to utilize models that allow multiple neuron firings^46,47^ combined with the SSR methods.

Previous studies have developed methods that suppress the firing of SNNs in the framework of the surrogate gradient method^27–29^. They applied direct regularization to the spike variable $s(t)\in \{0, 1\}$ represented at each time step to the time-discretized SNN. The gradient calculation is made possible using the surrogate function $\frac{d s(t)}{dv(t)}=\sigma (v(t))$^18^. This method is closely related to the M-SSR proposed in this paper. In the surrogate gradient method, the spike variables above are treated as a function of the membrane potential $s(t)=\int _{-\infty } ^{v(t)} \sigma (v') dv'$. In this sense, the idea is similar to the loss in Eq. (2), which integrates the membrane potential. By contrast, M-SSR, unlike the previous method^27–29^, can be transformed from the time-integration form to the timing form by setting ${\hat{v}}\rightarrow V_\text {th}$. This may correspond to the fact that the learning method with the surrogate function can transition to a timing-like learning method by taking a limit^20^. Interestingly, as shown in Figs. 3 and 4, the sparsity–accuracy property improves as ${\hat{v}}$ gets closer to $V_\text {th}$. This suggests that timing-based sparse regularization is more effective in timing-based learning. We note that F-SSR is a regularization method using firing conditions, which is unique to timing-based learning.

SNNs can operate efficiently on neuromorphic hardware^28,48^. Because the energy consumed by the spike transmission increases as the firing frequency increases, reducing the firing frequency is an important issue in real-world applications^49^. SNNs with TTFS coding are expected to provide significant power advantages in hardware implementation due to their extremely sparse firing^36,37,50^. Several research groups have reported hardware implementations of such SNNs^39,40,51^. The SSR methods are expected to further improve the energy efficiency of SNNs. Moreover, unlike the methods in^27–29^, the SSR methods can calculate the gradient without observing the membrane potential, which may simplify the learning system on hardware. Finally, in addition to the reduction in the firing rate, the combination of binarized weights^52^ and pruned weights^29,53,54^ is expected to make the SNN model more suitable for hardware implementation.

In this paper, we employed SNNs wherein spikes represent all-or-none binary signals^55^. Nevertheless, there exists physiological evidence suggesting that spikes can transmit information in an analog manner, particularly observed within certain brain regions^13,56,57^. The analog characteristics of spikes can enhance specific information processing within the brain^13^. We firmly believe that the integration of digital-spike and analog-spike systems, possibly through a fusion of SNNs and ANNs, holds significant importance in the construction of brain-scale neuromorphic systems.

## Method

### SNN models

In this study, we constructed a multilayer SNN using the following LIF neuron model:

9 $$\begin{aligned} \frac{d}{dt} v_i ^{(l)} (t)&= \frac{1}{\tau _v} v_i ^{(l)} (t) + I_{i}^{(l)} , \end{aligned}$$

10 $$\begin{aligned} \frac{d}{dt} I_i ^{(l)} (t)&=\frac{1}{\tau _I} I_i ^{(l)} (t) + \sum _{j=1} ^{N^{(l-1)}} w_{ij}^{(l)} \delta (t - t_j ^{(l-1)}), \end{aligned}$$

where $v_i^{(l)}$ is the membrane potential of neuron *i* in the *l*th layer, $I_i^{(l)}$ is the synaptic current input to the neuron, $w_{ij}^{(l)}$ is the coupling strength from neuron *j* in the $l-1$th layer to neuron *i* in the *l*th layer, and $t_j^{(l-1)}$ is the firing time of neuron *j* in the $l-1$th layer. $\delta$ is Dirac delta function. Furthermore, $\tau _v$ is the time constant of the membrane potential and $\tau _I$ is the time constant of the synaptic current. $N^{(l)}$ is the number of neurons that make up the *l*th layer. Neurons fire when the membrane potential reaches the firing threshold $V_\text {th}$ and generate spikes. After firing, the membrane potential is fixed at 0 and never fires again. The membrane potential of the model described in Eq. (9) is analytically obtained as follows:

11 $$\begin{aligned} v_i^{(l)}(t)&= \frac{\tau _v \tau _I}{\tau _v - \tau _I} \sum _{j=1}^{N^{(l-1)}} w_{ij}^{(l)}\kappa (t -t_j^{(l-1)}), \end{aligned}$$

12 $$\begin{aligned} \kappa (t)&= \theta (t) \left[ \exp \left( -\frac{t}{\tau _v}\right) - \exp \left( - \frac{t}{\tau _I}\right) \right] , \end{aligned}$$

13 $$\begin{aligned} \theta (t)&= {\left\{ \begin{array}{ll} 0, \text { for } t<0, \\ 1, \text { for } 0\le t. \end{array}\right. } \end{aligned}$$

The experiments in this study consider the three cases $(\tau _v, \tau _I) \in \{ (2\tau , \tau ), (\infty , \tau )$, and $(\infty , \infty )\}$. Note that the learning characteristics of SNNs with TTFS coding were investigated for the cases of $\tau _v= \tau _I = \infty$^36^, $\tau _v=\infty$^33^, and $\tau _v \ne \infty ,\tau _I \ne \infty$^35,39^.

The firing time in each case can be calculated from the condition $v_i^{(l)}(t_i^{(l)})=V_\text {th}$ as follows:

14 $$\begin{aligned} t_i^{(l)} = {\left\{ \begin{array}{ll} \frac{V_\text {th} + \sum _{j\in \Gamma _i^{(l)}} w_{ij}^{(l)}t_j^{(l)}}{\sum _{j\in \Gamma _i^{(l)}} w_{ij}^{(l)}}, \text { for } (\tau _v, \tau _I)=(\infty , \infty ),\\ \tau \ln \left[ \frac{\sum _{j\in \Gamma _i^{(l)}} w_{ij}^{(l)} \exp \left( \frac{t_j^{(l-1)}}{\tau } \right) }{\sum _{j\in \Gamma _i^{(l)}} w_{ij}^{(l)} - V_\text {th}\tau ^{-1}}\right] , \text { for }(\tau _v, \tau _I)=(\infty , \tau ),\\ 2\tau \ln \left[ \tau \frac{b_i^{(l)} - \sqrt{(b_i^{(l)})^2 - 2a_i^{(l)}\tau ^{-1}V_\text {th}}}{V_\text {th}} \right] , \text { for }(\tau _v, \tau _I)=(2\tau , \tau ), \end{array}\right. } \end{aligned}$$

where $\Gamma _i^{(l)}$ denotes the index set of spikes input to the *l*th layer neuron *i* up to firing time $t_i^{(l)}$. We define the following variables:

15 $$\begin{aligned} a_i^{(l)}= \sum _{j\in \Gamma _i^{(l)}} w_{ij}^{(l)} \exp \left( \frac{t_j^{(l-1)}}{\tau } \right) ,~ b_i^{(l)}= \sum _{j\in \Gamma _i^{(l)}} w_{ij}^{(l)} \exp \left( \frac{t_j^{(l-1)}}{2\tau } \right) . \end{aligned}$$

A detailed derivation of the firing time in the case of $(\tau _v, \tau _I) = (\tau , 2\tau )$ is given in the Supplementary Material.

### Learning algorithms

Supervised learning of the SNN was performed using the following cost function

16 $$\begin{aligned} C&= L(t^{(M)}) + \gamma _1 T(t^{(M)}) + \gamma _2 V + \gamma _3 Q, \end{aligned}$$

17 $$\begin{aligned} L&= \sum _{i=1}^{N^{(M)}} \kappa _i \ln S_i, \end{aligned}$$

18 $$\begin{aligned} S_i&= \frac{\exp \left( \frac{t_i^{(M)}}{\tau _\text {soft}}\right) }{\sum _{j=1}^{N^{(M)}} \exp \left( \frac{t_j^{(M)}}{\tau _\text {soft}}\right) }, \end{aligned}$$

19 $$\begin{aligned} T&= \sum _{i=1}^{N^{(M)}}\left( t_i^{(M)} - t^\text {ref}\right) ^2, \end{aligned}$$

where *M* represents the output layer and $t^{(M)}=\left( t_1^{(M)}, t_2^{(M)}, \dots , t_{N^{(M)}}^{(M)}\right)$. The value of the teacher label $\kappa _i$ is equal to one when the *i*th label is assigned and zero otherwise. Parameters $\gamma _1$, $\gamma _2$, and $\gamma _3$ are real numbers, and they respectively control the significance of the temporal penalty term *T*^36^, the membrane potential loss *V* (Eq. 1), and the firing condition term *Q* (Eq. 7). Parameter $\tau _\text {soft}$ is a positive real number, which adjusts the softmax scaling. Learning was performed by minimizing this cost function using the gradient method with the Adam optimizer^58^ at a learning rate of $\eta$. On the CIFAR-10 task, the coefficient $\gamma _3$ was set to a negative number to promote firing. In this case, the firing condition term *Q* was modified as follows:

20 $$\begin{aligned} Q_i^{(l)}&= {\left\{ \begin{array}{ll} \sum _{i=1}^{N^{(l-1)}} w_{ij}^{(l)}, ~\text {if not fired}, \\ 0,~\text {otherwise.} \\ \end{array}\right. } \end{aligned}$$

We promoted the neurons to fire only when the corresponding neurons did not fire.

### Dataset

The MNIST, Fashon-MNIST, and CIFAR-10 datasets include 2-dimensional image data. In the MNIST and Fashion-MNIST datasets, each image has one channel, whereas in the CIFAR-10 dataset, the images have three channels. To process such image data, we first normalized the pixel intensity to [0, 1]. Then, we obtained an input spike as follows:

21 $$\begin{aligned} t_{ijk}^{(0)} = \tau _\text {in} (1-x_{ijk}), \end{aligned}$$

where $x_{ijk}$ is the normalized pixel intensity, the first and second indices represent the coordinates of the pixel, and the third index represents the channel number. Here, $\tau _\text {in}$ is a positive constant. We set $\tau _\text {in}=5$ in all experiments. When spikes are input to a fully connected layer, the input tensors are reshaped into one-dimensional tensors. For the CIFAR-10 dataset, to avoid the problem of the first hidden layer firing too early and ignoring later inputs, the number of channels was doubled as follows:

22 $$\begin{aligned} x_{i,j,k} = 1 - x_{i,j,k-3}~(k=3,4,5). \end{aligned}$$

Furthermore, we used data augmentation (horizontal flipping, rotation, and cropping) as in the previous study^45^.

### Supplementary Information

Supplementary Information.

## Data Availability

The datasets utilized in this study are publicly accessible^42–44^. To access them, you have the option to use tools such as PyTorch for downloading purposes. Alternatively, you can opt for manual downloads directly from the following website: http://yann.lecun.com/exdb/mnist/ (MNIST), https://github.com/zalandoresearch/fashion-mnist (Fashion-MNIST), and https://www.cs.toronto.edu/~kriz/cifar.html (CIFAR-10).

## Code availability

Computer codes are available from Y.S. upon request.

## References

[1] Roy K, Jaiswal A, Panda P (2019). Towards spike-based machine intelligence with neuromorphic computing. Nature.

[2] Guo W, Fouda ME, Eltawil AM, Salama KN (2021). Neural coding in spiking neural networks: A comparative study for robust neuromorphic systems. Front. Neurosci..

[3] Auge D, Hille J, Mueller E, Knoll A (2021). A survey of encoding techniques for signal processing in spiking neural networks. Neural Process. Lett..

[4] Diehl, P. U., Neil, D., Binas, J., Cook, M., Liu, S., & Pfeiffer, M. Fast-classifying, high-accuracy spiking deep networks through weight and threshold balancing. In *2015 International Joint Conference on Neural Networks (IJCNN)*. pp. 1–8 (2015).

[5] Rueckauer B, Lungu I-A, Yuhuang H, Pfeiffer M, Liu S-C (2017). Conversion of continuous-valued deep networks to efficient event-driven networks for image classification. Front. Neurosci..

[6] Kim S, Park S, Na B, Yoon S (2020). Spiking-YOLO: Spiking neural network for energy-efficient object detection. Proc. AAAI Conf. Artif. Intell..

[7] Davies M (2018). Loihi: A neuromorphic manycore processor with on-chip learning. IEEE Micro.

[8] Barth AL, Poulet JFA (2012). Experimental evidence for sparse firing in the neocortex. Trends in Neurosci..

[9] Fujii H, Ito H, Aihara K, Ichinose N, Tsukada M (1996). Dynamical cell assembly hypothesis—Theoretical possibility of spatio-temporal coding in the cortex. Neural Netw..

[10] Gollisch T, Meister M (2008). Rapid neural coding in the retina with relative spike latencies. Science.

[11] Portelli G, Barrett JM, Hilgen G, Masquelier T, Maccione A, DiMarco S, Berdondini L, Kornprobst P, Sernagor E (2016). Rank order coding: A retinal information decoding strategy revealed by large-scale multielectrode array retinal recordings. eNeuro.

[12] Jaramillo J, Kempter R (2017). Phase precession: A neural code underlying episodic memory?. Curr. Opin. Neurobiol..

[13] Zbili M, Rama S, Yger P, Inglebert Y, Boumedine-Guignon N, Fronzaroli-Moliniere L, Brette R, Russier M, Debanne D (2020). Axonal Na^+^ channels detect and transmit levels of input synchrony in local brain circuits. Sci. Adv..

[14] Tavanaei A, Ghodrati M, Kheradpisheh SR, Masquelier T, Maida A (2019). Deep learning in spiking neural networks. Neural Netw..

[15] Pfeiffer M, Pfeil T (2018). Deep learning with spiking neurons: Opportunities and challenges. Front. Neurosci..

[16] Dampfhoffer, M., Mesquida, T., Valentian, A. & Anghel, L. Backpropagation-based learning techniques for deep spiking neural networks: A survey. In*IEEE Transactions on Neural Networks and Learning Systems*. pp 1–16 (2023). 10.1109/TNNLS.2023.3263008 37027264

[17] Eshraghian, J.K., Ward, M., Neftci, E.,Wang, X., Lenz, G., Dwivedi, G., Bennamoun, M., Jeong, D.S. & Lu, W.D. Training spiking neural networks using lessons from deep learning. arXiv:2109.12894 (2023).

[18] Neftci EO, Mostafa H, Zenke F (2019). Surrogate gradient learning in spiking neural networks: Bringing the power of gradient-based optimization to spiking neural networks. IEEE Signal Process. Mag..

[19] Neftci EO, Augustine C, Paul S, Detorakis G (2017). Event-driven random back-propagation: Enabling neuromorphic deep learning machines. Front. Neurosci..

[20] Huh, D. & Sejnowski, T.J. Gradient descent for spiking neural networks. In *Proceedings of the 32nd International Conference on Neural Information Processing Systems*. pp. 1440–1450 (2018).

[21] Zenke F, Ganguli S (2018). SuperSpike: Supervised learning in multilayer spiking neural networks. Neural Comput..

[22] Yujie W, Deng L, Li G, Zhu J, Shi L (2018). Spatio-temporal backpropagation for training high-performance spiking neural networks. Front. Neurosci..

[23] Zenke F, Vogels TP (2021). The remarkable robustness of surrogate gradient learning for instilling complex function in spiking neural networks. Neural Comput..

[24] Yin B, Corradi F, Bohté SM (2021). Accurate and efficient time-domain classification with adaptive spiking recurrent neural networks. Nat. Mach. Intell..

[25] Bellec G, Scherr F, Subramoney A, Hajek E, Salaj D, Legenstein R, Maass W (2020). A solution to the learning dilemma for recurrent networks of spiking neurons. Nat. Commun..

[26] Kim Y, Panda P (2021). Revisiting batch normalization for training low-latency deep spiking neural networks from scratch. Front. Neurosci..

[27] Pellegrini, T., Zimmer, R., & Masquelier, T. Low-activity supervised convolutional spiking neural networks applied to speech commands recognition. In *2021 IEEE Spoken Language Technology Workshop (SLT)*. pp. 97–103 (2021).

[28] Cramer B, Billaudelle S, Kanya S, Leibfried A, Grübl A, Karasenko V, Pehle C, Schreiber K, Stradmann Y, Weis J, Schemmel J, Zenke F (2022). Surrogate gradients for analog neuromorphic computing. Proc. Natl. Acad. Sci..

[29] Yan Y, Chu H, Jin Y, Huan Y, Zou Z, Zheng L (2022). Backpropagation with sparsity regularization for spiking neural network learning. Front. Neurosci..

[30] Bohte SM, Kok JN, La Poutré H (2002). Error-backpropagation in temporally encoded networks of spiking neurons. Neurocomputing.

[31] Thorpe S, Delorme A, Van Rullen R (2001). Spike-based strategies for rapid processing. Neural Netw..

[32] Bonilla L, Gautrais J, Thorpe S, Masquelier T (2022). Analyzing time-to-first-spike coding schemes: A theoretical approach. Front. Neurosci..

[33] Mostafa H (2018). Supervised learning based on temporal coding in spiking neural networks. IEEE Trans. Neural Netw. Learn. Syst..

[34] Kheradpisheh SR, Masquelier T (2020). Temporal backpropagation for spiking neural networks with one spike per neuron. Int. J. Neural Syst..

[35] Comşa I-M, Potempa K, Versari L, Fischbacher T, Gesmundo A, Alakuijala J (2022). Temporal coding in spiking neural networks with alpha synaptic function: Learning with backpropagation. IEEE Trans. Neural Netw. Learn. Syst..

[36] Sakemi Y, Morino K, Morie T, Aihara K (2023). A supervised learning algorithm for multilayer spiking neural networks based on temporal coding toward energy-efficient vlsi processor design. IEEE Trans. Neural Netw. Learn. Syst..

[37] Sakemi, Y., Morie, T., Hosomi, T. & Aihara, K. Effects of VLSI circuit constraints on temporal-coding multilayer spiking neural networks. arXiv:2106.10382 (2021).

[38] Zhang M, Wang J, Jibin W, Belatreche A, Amornpaisannon B, Zhang Z, Miriyala VPK, Hong Q, Chua Y, Carlson TE, Li H (2022). Rectified linear postsynaptic potential function for backpropagation in deep spiking neural networks. IEEE Trans. Neural Netw. Learn. Syst..

[39] Göltz J (2021). Fast and energy-efficient neuromorphic deep learning with first-spike times. Nat. Mach. Intell..

[40] Oh S, Kwon D, Yeom G, Kang W-M, Lee S, Woo SY, Kim J, Lee J-H (2022). Neuron circuits for low-power spiking neural networks using time-to-first-spike encoding. IEEE Access.

[41] Sarwar Murshed MG, Murphy C, Hou D, Khan N, Ananthanarayanan G, Hussain F (2021). Machine learning at the network edge: A survey. ACM Comput. Surv..

[42] LeCun Y, Bottou L, Bengio Y, Haffner P (1998). Gradient-based learning applied to document recognition. Proc. IEEE.

[43] Xiao, H., Rasul, K. & Vollgraf, R. Fashion-MNIST: A novel image dataset for benchmarking machine learning algorithms. arXiv:1708.07747 (2017).

[44] Krizhevsky, A. *Learning Multiple Layers of Features from Tiny Images*.

[45] Zhou S, Li X, Chen Y, Chandrasekaran ST, Sanyal A (2021). Temporal-coded deep spiking neural network with easy training and robust performance. Proc. AAAI Conf. Artif. Intell..

[46] Wunderlich TC, Pehle C (2021). Event-based backpropagation can compute exact gradients for spiking neural networks. Sci. Rep..

[47] Yamamoto, K., Sakemi, Y., & Aihara, K. Timing-based backpropagation in spiking neural networks without single-spike restrictions. arXiv:2211.16113 (2022).

[48] Moradi S, Qiao N, Stefanini F, Indiveri G (2018). A scalable multicore architecture with heterogeneous memory structures for dynamic neuromorphic asynchronous processors (DYNAPs). IEEE Trans. Biomed. Circuits Syst..

[49] Rançon U, Cuadrado-Anibarro J, Cottereau BR, Masquelier T (2022). Stereospike: Depth learning with a spiking neural network. IEEE Access.

[50] Sakemi, Y., Morino, K., Morie, T., Hosomi, T. & Aihara, K. A spiking neural network with resistively coupled synapses using time-to-first-spike coding towards efficient charge-domain computing. In *2022 IEEE International Symposium on Circuits and Systems (ISCAS)*. 2152–2156 (2022).

[51] Oh, S., Kwon, D., Yeom, G., Kang, W.-M., Lee, S., Woo, S.Y., Kim, J.S., Park, M.K. & Lee, J.-H. Hardware implementation of spiking neural networks using time-to-first-spike encoding. arXiv:2006.05033 (2020).

[52] Kheradpisheh SR, Mirsadeghi M, Masquelier T (2022). BS4NN: Binarized spiking neural networks with temporal coding and learning. Neural Process. Lett..

[53] Faghihi F, Alashwal H, Moustafa AA (2022). A synaptic pruning-based spiking neural network for hand-written digits classification. Front. Artif. Intell..

[54] Han, B., Zhao, F., Zeng, Y., & Pan, W. Adaptive sparse structure development with pruning and regeneration for spiking neural networks. arXiv preprint arXiv:2211.12219 (2022).

[55] Gerstner W, Kistler WM, Naud R, Paninski L (2014). Neuronal Dynamics: From Single Neurons to Networks and Models of Cognition.

[56] Clark B, Häusser M (2006). Neural coding: Hybrid analog and digital signalling in axons. Curr. Biol..

[57] Brunner J, Szabadics J (2016). Analogue modulation of back-propagating action potentials enables dendritic hybrid signalling. Nat. Commun..

[58] Kingma, D.P. & Ba, J. Adam: A method for stochastic optimization. arXiv:1412.6980 (2014).

