# Supplementary material for: Sparse-firing regularization methods for spiking neural networks with time-to-first-spike coding
Source: Sci Rep. 2023 Dec 21;13:22897. doi: 10.1038/s41598-023-50201-5 (PMC10739753; doi:10.1038/s41598-023-50201-5)
Supplement: Supplementary file 1 — Supplementary Information. [file 41598_2023_50201_MOESM1_ESM.pdf]

Supplementary materials for: Sparse-Firing Regularization Methods for Spiking Neural Networks with Time-to-First Spike Coding

Yusuke Sakemi[1,2,*], Kakei Yamamoto[3], Takeo Hosomi[4], and Kazuyuki Aihara[1,2]

[1]Research Center for Mathematical Engineering, Chiba Institute of Technology, Narashino, Japan
[2]International Research Center for Neurointelligence (WPI-IRCN), The University of Tokyo, Tokyo, Japan
[3]Massachusetts Institute of Technology, Cambridge, USA
[4]NEC Corporation, Kawasaki, Japan
[*]Corresponding author

December 7, 2023

Derivation of M-SSR

In this section, we present a detailed derivation of M-SSR. If $\hat{v}$ is sufficiently close to V_{th}, $\hat{t}_i^{(l)}$, the time at which the membrane potential is $\hat{v}$ can be assumed to be a single point. Moreover, we can assume that the number of input spikes can be assumed to be constant $\left(\Gamma_i^{(l)}\right)$ in the time interval $[\hat{t}_i^{(l)}, t_i^{(l)}]$. From the above, the integral-form loss can be transformed as follows

$$V_i^{(l)} = \frac{1}{V_{\mathrm{th}} - \hat{v}} \int_0^T dt \left(v_i^{(l)}(t) - \hat{v}\right) \theta \left(v_i^{(l)}(t) - \hat{v}\right) \theta \left(t_i - t\right), \tag{1}$$

$$= \frac{1}{V_{\mathrm{th}} - \hat{v}} \int_{\hat{t}_i^{(l)}}^{t_i^{(l)}} dt \left(v_i^{(l)}(t) - \hat{v}\right). \tag{2}$$

Note that $V_i^{(l)} = 0$ if the neuron does not fire. Importantly, when computing the gradient of this integral, the integration range $[\hat{t}, t_i^{(l)}]$ should be fixed. This is because if they are not treated as fixed values, the SNNs learn to make the membrane potential rise rapidly, thus the effect of firing suppression cannot be obtained. We can omit the constant term $\hat{v}(t_i^{(l)} - \hat{t}_i^{(l)})$, which is not involved in the learning process, and we only need to calculate the following:

$$V_i = \frac{1}{V_{\mathrm{th}} - \hat{v}} \int_{\hat{t}_i^{(l)}}^{t_i^{(l)}} v_i^{(l)}(t) dt. \tag{3}$$

In the following, the limit of the above integral ($\hat{v} \to V_{\mathrm{th}}$) is calculated for various neuron models ($(\tau_v, \tau_I) = (\infty, \infty), (\infty, \tau), \text{and } (2\tau, \tau)$).

Neuron model with $\tau_v = \infty$ and $\tau_I = \infty$

When $\tau_I = \tau_v = \infty$, the membrane potential and firing time can be calculated as follows:

$$v_i^{(l)}(t) = \sum_{j=1}^{N^{(l-1)}} w_{ij}^{(l)} (t - t_j^{(l-1)}) \theta(t - t_j^{(l-1)}), \tag{4}$$

$$t_i^{(l)} = \frac{V_{\text{th}} + \sum_{j \in \Gamma_i^{(l)}} w_{ij}^{(l)} t_j^{(l)}}{\sum_{j \in \Gamma_i^{(l)}} w_{ij}^{(l)}}, \tag{5}$$

$$\hat{t}_i^{(l)} = \frac{\hat{v} + \sum_{j \in \Gamma_i^{(l)}} w_{ij}^{(l)} t_j^{(l)}}{\sum_{j \in \Gamma_i^{(l)}} w_{ij}^{(l)}}, \tag{6}$$

$$\frac{t_i^{(l)} - \hat{t}_i^{(l)}}{V_{\text{th}} - \hat{v}} = \frac{1}{\sum_{j \in \Gamma_i^{(l)}} w_{ij}}. \tag{7}$$

Using these equations, the limit ($\hat{v} \to V_{\text{th}}$) can be calculated as follows:

$$V_i^{(l)} = \frac{1}{V_{\text{th}} - \hat{v}} \int_{\hat{t}_i^{(l)}}^{t_i^{(l)}} dt v_i^{(l)}(t) \tag{8}$$

$$= \frac{1}{V_{\text{th}} - \hat{v}} \int_{\hat{t}_i^{(l)}}^{t_i^{(l)}} dt \sum_{j \in \Gamma_i^{(l)}} w_{ij}^{(l)} \left(t - t_j^{(l-1)}\right) \tag{9}$$

$$= \frac{1}{V_{\text{th}} - \hat{v}} \left[\frac{1}{2} \sum_{j \in \Gamma_i^{(l)}} w_{ij}^{(l)} \left(t - t_j^{(l-1)}\right)^2 \right]_{\hat{t}_i^{(l)}}^{t_i^{(l)}} \tag{10}$$

$$= \frac{1}{2\left(V_{\text{th}} - \hat{v}\right)} \sum_{j \in \Gamma_i^{(l)}} w_{ij}^{(l)} \left[\left({\color{blue} t_i^{(l)}} - t_j^{(l-1)} \right)^2 - \left({\color{blue} \hat{t}_i^{(l)}} - t_j^{(l-1)} \right)^2 \right] \tag{11}$$

$$= \frac{1}{2} \frac{{\color{blue} t_i^{(l)}} - {\color{blue} \hat{t}_i^{(l)}}}{V_{\text{th}} - \hat{v}} \sum_{j \in \Gamma_i^{(l)}} w_{ij} \left({\color{blue} t_i^{(l)}} + {\color{blue} \hat{t}_i^{(l)}} - 2 t_j^{(l-1)} \right) \tag{12}$$

$$\overset{\text{Eq.(7)}}{=} \frac{1}{{\color{blue} \sum_{j \in \Gamma_i^{(l)}} w_{ij}}} \left[{\color{blue} t_i^{(l)}} \sum_j w_{ij} - \sum_j w_{ij} t_j^{(l-1)} \right]. \tag{13}$$

Note that the blue variables are related to the integral range, which is treated as a fixed value when calculating the gradient, as mentioned above.

Neuron model with $\tau_v = \infty$ and $\tau_I = \tau$

When $\tau_v = \infty$ and $\tau_I = \tau$, the membrane potential and firing time are given by [1]

$$v_i^{(l)}(t) = \tau \sum_{j=1}^{N^{(l-1)}} w_{ij}^{(l)} \theta(t - t_j^{(l-1)}) \left[1 - \exp\left(- \frac{t - t_j^{(l-1)}}{\tau} \right) \right], \tag{14}$$

$$t_i^{(l)} = \tau \ln \left[\frac{\sum_{j \in \Gamma_i^{(l)}} w_{ij}^{(l)} \exp\left(\frac{t_j^{(l-1)}}{\tau} \right)}{\sum_{j \in \Gamma_i^{(l)}} w_{ij}^{(l)} - V_{\text{th}} \tau^{-1}} \right], \tag{15}$$

$$\hat{t}_i^{(l)} = \tau \ln \left[\frac{\sum_{j \in \Gamma_i^{(l)}} w_{ij}^{(l)} \exp\left(\frac{t_j^{(l-1)}}{\tau} \right)}{\sum_{j \in \Gamma_i^{(l)}} w_{ij}^{(l)} - \hat{v} \tau^{-1}} \right]. \tag{16}$$

We also obtain the following in $\hat{v} \to V_{\text{th}}$:

$$\frac{t_i^{(l)} - \hat{t}_i^{(l)}}{V_{\text{th}} - \hat{v}} = \frac{\tau}{V_{\text{th}} - \hat{v}} \ln \frac{\sum_{j \in \Gamma_i^{(l)}} w_{ij}^{(l)} \exp\left(\frac{t_j^{(l)}}{\tau}\right)}{\sum_{j \in \Gamma_i^{(l)}} w_{ij}^{(l)} - V_{\text{th}}\tau^{-1}} - \frac{\tau}{V_{\text{th}} - \hat{v}} \ln \frac{\sum_{j \in \Gamma_i^{(l)}} w_{ij}^{(l)} \exp\left(\frac{t_j^{(l)}}{\tau}\right)}{\sum_{j \in \Gamma_i^{(l)}} w_{ij}^{(l)} - \hat{v}\tau^{-1}} \tag{17}$$

$$= \frac{\tau}{V_{\text{th}} - \hat{v}} \ln \frac{\sum_{j \in \Gamma_i^{(l)}} w_{ij}^{(l)} - \hat{v}\tau^{-1}}{\sum_{j \in \Gamma_i^{(l)}} w_{ij}^{(l)} - V_{\text{th}}\tau^{-1}} \tag{18}$$

$$= \frac{\tau}{V_{\text{th}} - \hat{v}} \ln \left(1 + \frac{(V_{\text{th}} - \hat{v})\,\tau^{-1}}{\sum_{j \in \Gamma_i^{(l)}} w_{ij}^{(l)} - V_{\text{th}}\tau^{-1}}\right) \tag{19}$$

$$\overset{\hat{v} \to V_{\text{th}}}{=} \frac{1}{\sum_{j \in \Gamma_i^{(l)}} w_{ij}^{(l)} - V_{\text{th}}\tau^{-1}}. \tag{20}$$

Using these, the limit ($\hat{v} \to V_{\text{th}}$) can be calculated as follows:

$$\frac{\int dt v_i^{(l)}(t)}{V_{\text{th}} - \hat{v}} = \frac{\tau}{V_{\text{th}} - \hat{v}} \left[\sum_{j \in \Gamma_i^{(l)}} w_{ij}^{(l)} \left\{t + \tau \exp\left(\frac{t_j^{(l-1)}}{\tau}\right) \exp\left(-\frac{t}{\tau}\right)\right\}\right]_{\hat{t}_i^{(l)}}^{t_i^{(l)}} \tag{21}$$

$$= \frac{\tau}{V_{\text{th}} - \hat{v}} \sum_{j \in \Gamma_i^{(l)}} w_{ij}^{(l)} \left[(t_i^{(l)} - \hat{t}_i^{(l)}) + \tau \exp\left(\frac{t_j^{(l-1)}}{\tau}\right) \left\{\exp\left(-\frac{t_i^{(l)}}{\tau}\right) - \exp\left(-\frac{\hat{t}_i^{(l)}}{\tau}\right)\right\}\right] \tag{22}$$

$$= \frac{\tau(t_i^{(l)} - \hat{t}_i^{(l)})}{V_{\text{th}} - \hat{v}} \sum_{j \in \Gamma_i^{(l)}} w_{ij}^{(l)} \left[1 + \frac{\tau}{(t_i^{(l)} - \hat{t}_i^{(l)})} \exp\left(\frac{t_j^{(l-1)}}{\tau}\right) \exp\left(-\frac{t_i^{(l)}}{\tau}\right) \left\{1 - \exp\left(\frac{t_i^{(l)} - \hat{t}_i^{(l)}}{\tau}\right)\right\}\right] \tag{23}$$

$$\overset{\hat{v} \to V_{\text{th}}}{=} \tau \frac{1}{\sum_{j \in \Gamma_i^{(l)}} w_{ij}^{(l)} - V_{\text{th}}\tau^{-1}} \sum_{j \in \Gamma_i^{(l)}} w_{ij}^{(l)} \left\{1 - \exp\left(\frac{t_j^{(l)}}{\tau}\right) \exp\left(-\frac{t_i^{(l)}}{\tau}\right)\right\} \tag{24}$$

$$= \tau \frac{1}{\sum_{j \in \Gamma_i^{(l)}} w_{ij}^{(l)} - V_{\text{th}}\tau^{-1}} \left[\left(\sum_{j \in \Gamma_i^{(l)}} w_{ij}^{(l)}\right) - \exp\left(-\frac{t_i^{(l)}}{\tau}\right) \sum_{j \in \Gamma_i^{(l)}} w_{ij}^{(l)} \exp\left(\frac{t_j^{(l-1)}}{\tau}\right)\right]. \tag{25}$$

Alpha-synaptic neuron model with $\tau_v = 2\tau_I = 2\tau$

When $\tau_v = 2\tau_I = 2\tau$, the membrane potential is given by

$$v_i^{(l)}(t) = 2\tau \sum_{j \in \Gamma_i^{(l)}} w_{ij}^{(l)} \theta(t - t_j^{(l-1)}) \left[\exp\left(-\frac{t - t_j^{(l-1)}}{2\tau}\right) - \exp\left(-\frac{t - t_j^{(l-1)}}{\tau}\right)\right]. \tag{26}$$

From the firing condition $v_i^{(l)}\left(t_i^{(l)}\right) = V_{\text{th}}$, we obtain the following:

$$\exp\left(-\frac{t_i^{(l)}}{2\tau}\right) \sum_{j \in \Gamma_i^{(l)}} w_{ij}^{(l)} \exp\left(\frac{t_j^{(l-1)}}{2\tau}\right) - \exp\left(-\frac{t_i^{(l)}}{\tau}\right) \sum_{j \in \Gamma_i^{(l)}} w_{ij}^{(l)} \exp\left(\frac{t_j^{(l-1)}}{\tau}\right) = \frac{V_{\text{th}}}{2\tau} \tag{27}$$

$$\left[\exp\left(-\frac{t_i^{(l)}}{2\tau}\right)\right]^2 \sum_{j \in \Gamma_i^{(l)}} w_{ij}^{(l)} \exp\left(\frac{t_j^{(l-1)}}{\tau}\right) - \exp\left(-\frac{t_i^{(l)}}{2\tau}\right) \sum_{j \in \Gamma_i^{(l)}} w_{ij}^{(l)} \exp\left(\frac{t_j^{(l-1)}}{2\tau}\right) + \frac{V_{\text{th}}}{2\tau} = 0 \tag{28}$$

$$a_i \left[\exp\left(-\frac{t_i^{(l)}}{2\tau}\right)\right]^2 - b_i^{(l)} \exp\left(-\frac{t_i^{(l)}}{2\tau}\right) + \frac{V_{\text{th}}}{2\tau} = 0, \tag{29}$$

where we defined the following variables:

$$a_i^{(l)} = \sum_{j \in \Gamma_i^{(l)}} w_{ij}^{(l)} \exp\left(\frac{t_j^{(l-1)}}{\tau}\right), \ b_i^{(l)} = \sum_{j \in \Gamma_i^{(l)}} w_{ij}^{(l)} \exp\left(\frac{t_j^{(l-1)}}{2\tau}\right). \tag{30}$$

From the formula to solve a quadratic function, the firing time $t_i^{(l)}$ can be calculated from Eq. (29) as follows [2]:

$$\exp\left(-\frac{t_i^{(l)}}{2\tau}\right) = \frac{b_i^{(l)} + \sqrt{(b_i^{(l)})^2 - 2a_i^{(l)}\tau^{-1}V_{\text{th}}}}{2a_i^{(l)}} \tag{31}$$

$$t_i^{(l)} = -2\tau \ln\left[\frac{b_i^{(l)} + \sqrt{(b_i^{(l)})^2 - 2a_i^{(l)}\tau^{-1}V_{\text{th}}}}{2a_i^{(l)}}\right], \tag{32}$$

$$\tag{33}$$

where the other solution of the quadratic function is ignored because it indicates the time at which the membrane potential decreases from a value greater than V_{th} to a smaller value [2]. We also obtain the following:

$$\frac{t_i^{(l)} - \hat{t}_i^{(l)}}{V_{\text{th}} - \hat{v}} = \frac{2\tau}{V_{\text{th}} - \hat{v}} \ln \frac{b_i^{(l)} + \sqrt{(b_i^{(l)})^2 - 2a_i^{(l)}\tau^{-1}\hat{v}}}{b_i^{(l)} + \sqrt{(b_i^{(l)})^2 - 2a_i^{(l)}\tau^{-1}V_{\text{th}}}} \tag{34}$$

$$= \frac{2\tau}{V_{\text{th}} - \hat{v}} \ln \frac{b_i^{(l)} + \sqrt{(b_i^{(l)})^2 - 2a_i^{(l)}\tau^{-1}V_{\text{th}} + 2a_i^{(l)}\tau^{-1}\left(V_{\text{th}} - \hat{v}\right)}}{b_i^{(l)} + \sqrt{(b_i^{(l)})^2 - 2a_i^{(l)}\tau^{-1}V_{\text{th}}}} \tag{35}$$

$$\overset{\hat{v} \to V_{\text{th}}}{=} 2\tau \frac{a_i^{(l)}\tau^{-1}}{\left(b_i^{(l)} + \sqrt{(b_i^{(l)})^2 - 2a_i^{(l)}\tau^{-1}V_{\text{th}}}\right)\sqrt{(b_i^{(l)})^2 - 2a_i^{(l)}\tau^{-1}V_{\text{th}}}} \tag{36}$$

$$= \alpha_i^{(l)}. \tag{37}$$

We define the following variable:

$$\alpha_i^{(l)} = \frac{2a_i}{\left(b_i^{(l)} + \sqrt{(b_i^{(l)})^2 - 2a_i^{(l)}\tau^{-1}V_{\text{th}}}\right)\left(\sqrt{(b_i^{(l)})^2 - 2a_i^{(l)}\tau^{-1}V_{\text{th}}}\right)}. \tag{38}$$

Using these, the limit $(\hat{v} \to V_{\text{th}})$ can be calculated as follows:

$$\frac{\int v_i^{(l)}(t)dt}{V_{\text{th}} - \hat{v}} = \frac{2\tau}{V_{\text{th}} - \hat{v}} \sum_{j \in \Gamma_i^{(l)}} \left[w_{ij} \left\{ -2\tau \exp\left(-\frac{t - t_j^{(l)}}{2\tau} \right) + \tau \exp\left(-\frac{t - t_j^{(l-1)}}{\tau} \right) \right\} \right]_{\hat{t}_i^{(l)}}^{t_i^{(l)}} \tag{39}$$

$$= \frac{2\tau^2}{V_{\text{th}} - \hat{v}} \sum_{j \in \Gamma_i^{(l)}} w_{ij}^{(l)} \left[-2\exp\left(\frac{t_j^{(l-1)}}{2\tau} \right) \exp\left(-\frac{t}{2\tau} \right) + \exp\left(\frac{t_j^{(l-1)}}{\tau} \right) \exp\left(-\frac{t}{\tau} \right) \right]_{\hat{t}_i^{(l)}}^{t_i^{(l)}} \tag{40}$$

$$= \frac{2\tau^2}{V_{\text{th}} - \hat{v}} \sum_{j \in \Gamma_i^{(l)}} w_{ij}^{(l)} \left[-2\exp\left(\frac{t_j^{(l-1)}}{2\tau} \right) \left\{ \exp\left(-\frac{t_i^{(l)}}{2\tau} \right) - \exp\left(-\frac{\hat{t}_i^{(l)}}{2\tau} \right) \right\} \right.$$
$$\left. + \exp\left(\frac{t_j^{(l-1)}}{\tau} \right) \left\{ \exp\left(-\frac{t_i^{(l)}}{\tau} \right) - \exp\left(-\frac{\hat{t}_i^{(l)}}{\tau} \right) \right\} \right] \tag{41}$$

$$= \frac{2\tau^2 \left(t_i^{(l)} - \hat{t}_i^{(l)} \right)}{V_{\text{th}} - \hat{v}} \sum_{j \in \Gamma_i^{(l)}} \frac{w_{ij}^{(l)}}{t_i^{(l)} - \hat{t}_i^{(l)}} \left[-2\exp\left(\frac{t_j^{(l-1)}}{2\tau} \right) \exp\left(-\frac{t_i^{(l)}}{2\tau} \right) \left\{ 1 - \exp\left(\frac{t_i^{(l)} - \hat{t}_i^{(l)}}{2\tau} \right) \right\} \right.$$
$$\left. + \exp\left(\frac{t_j^{(l-1)}}{\tau} \right) \exp\left(-\frac{t_i^{(l)}}{\tau} \right) \left\{ 1 - \exp\left(\frac{t_i^{(l)} - \hat{t}_i^{(l)}}{\tau} \right) \right\} \right] \tag{42}$$

$$\overset{\hat{v} \to V_{\text{th}}}{=} 2\tau \alpha_i^{(l)} \left[\exp\left(-\frac{t_i^{(l)}}{2\tau} \right) \sum_{j \in \Gamma_i^{(l)}} w_{ij}^{(l)} \exp\left(\frac{t_j^{(l-1)}}{2\tau} \right) - \exp\left(-\frac{t_i^{(l)}}{\tau} \right) \sum_{j \in \Gamma_i^{(l)}} w_{ij}^{(l)} \exp\left(\frac{t_j^{(l-1)}}{\tau} \right) \right] \tag{43}$$

$$= 2\tau \alpha_i^{(l)} \left[\exp\left(-\frac{t_i^{(l)}}{2\tau} \right) b_i^{(l)} - \exp\left(-\frac{t_i^{(l)}}{\tau} \right) a_i^{(l)} \right]. \tag{44}$$

Numerical convergence analysis

M-SSR was derived by taking the limit of $\hat{v} \to V_{\text{th}}$ for the integral-form regularization. In this section, we confirm that the gradient of the integral-form regularization converges to that of M-SSR when $\hat{v}$ approaches V_{th} through numerical simulation. The Iris dataset [3] was used as input data. The Iris dataset consists of 150 instances of three-class data with four features. To process the data with an SNN, each feature was normalized to $[0, 1]$ and each element of the vector (x_i) was transformed to the time $t_i^{(0)} = \tau_{\text{in}} x_i$ $(i = 0, 1, 2, 3)$ of the input layer spikes, where $\tau_{\text{in}} = 5$. We also introduced a bias spike $t_4^{(0)} = 0$. This input spike was input to an SNN with two hidden layers (5-10-10-3). The cost function was active only for V, with $\gamma_2 = 1$. The error for the gradient of the weights for each layer was defined as follows:

$$Error^{(l)}(x) = \frac{1}{N^{(l)} N^{(l-1)}} \sum_{i=1}^{N^{(l)}} \sum_{j=1}^{N^{(l-1)}} \frac{\left| \frac{\partial C(\hat{v}=x)}{\partial w_{ij}^{(l)}} - \frac{\partial C(\hat{v} \to V_{\text{th}})}{\partial w_{ij}^{(l)}} \right|}{\left| \frac{\partial C(\hat{v}=x)}{\partial w_{ij}^{(l)}} \right|}. \tag{45}$$

The integral-form regularization was computed by setting $T = t^{\text{ref}}$ and dividing the integral by the time step width $\Delta t = \frac{T}{N_{\text{steps}}}$.

Figure 1 shows the results of the gradient error for various neuron models. The gradient error was calculated by averaging over the 150 data. For all neuron models and N_{steps}, we can confirm that the errors decrease as $\hat{v}$ is brought closer to V_{th}, and then the errors tend to increase. This increase in error is due to the fact that if $\hat{v}$ is too close to V_{th}, the integration range becomes extremely narrow, making it difficult to evaluate the integral. As N_{steps} is increased, it is found that the integrals can be evaluated more precisely up to the point where $\hat{v}$ is closer to V_{th}. As N_{steps} is increased, the minimum value of the gradient error decreases uniformly, and it is found to decrease to about 0.1% for $N_{\text{steps}} = 10^7$.

Effects of ξ

In the proposed SSR methods, the regularization loss is larger in the latter layers as the coefficient ξ increases, resulting in sparse firing characteristics in the latter layers. Figure 2 shows the effect of the value of ξ on the sparsity–accuracy tradeoff with M-SSR and F-SSR. We trained the SNNs with three hidden layers (784-400-400-400-10), and the results are shown for the MNIST and Fashion-MNIST datasets. For each dataset, the top three panels show the sparsity–accuracy tradeoffs for the first, second, and third hidden layers, and the bottom panel shows the average results for all hidden layers. In the case of M-SSR, when $p_{\text{layer}} = 1$, the latter layer is not sparse, but when $p_{\text{layer}} = 6$, both layers are sparse. By contrast, in the case of F-SSR, even when $p_{\text{layer}} = 1$, the latter layers are relatively sparse. This difference can be attributed to the fact that in M-SSR, the regularization error propagates backward to the previous layer, whereas in F-SSR, the regularization error is local and does not propagate backward.

References

[1] H. Mostafa. Supervised learning based on temporal coding in spiking neural networks. *IEEE Transactions on Neural Networks and Learning Systems*, 29(7):3227–3235, 2018.

[2] J Göltz et al. Fast and energy-efficient neuromorphic deep learning with first-spike times. *Nature Machine Intelligence*, 3:823–835, 2021.

[3] F. Pedregosa, G. Varoquaux, A. Gramfort, V. Michel, B. Thirion, O. Grisel, M. Blondel, P. Prettenhofer, R. Weiss, V. Dubourg, J. Vanderplas, A. Passos, D. Cournapeau, M. Brucher, M. Perrot, and E. Duchesnay. Scikit-learn: Machine learning in Python. *Journal of Machine Learning Research*, 12:2825–2830, 2011.

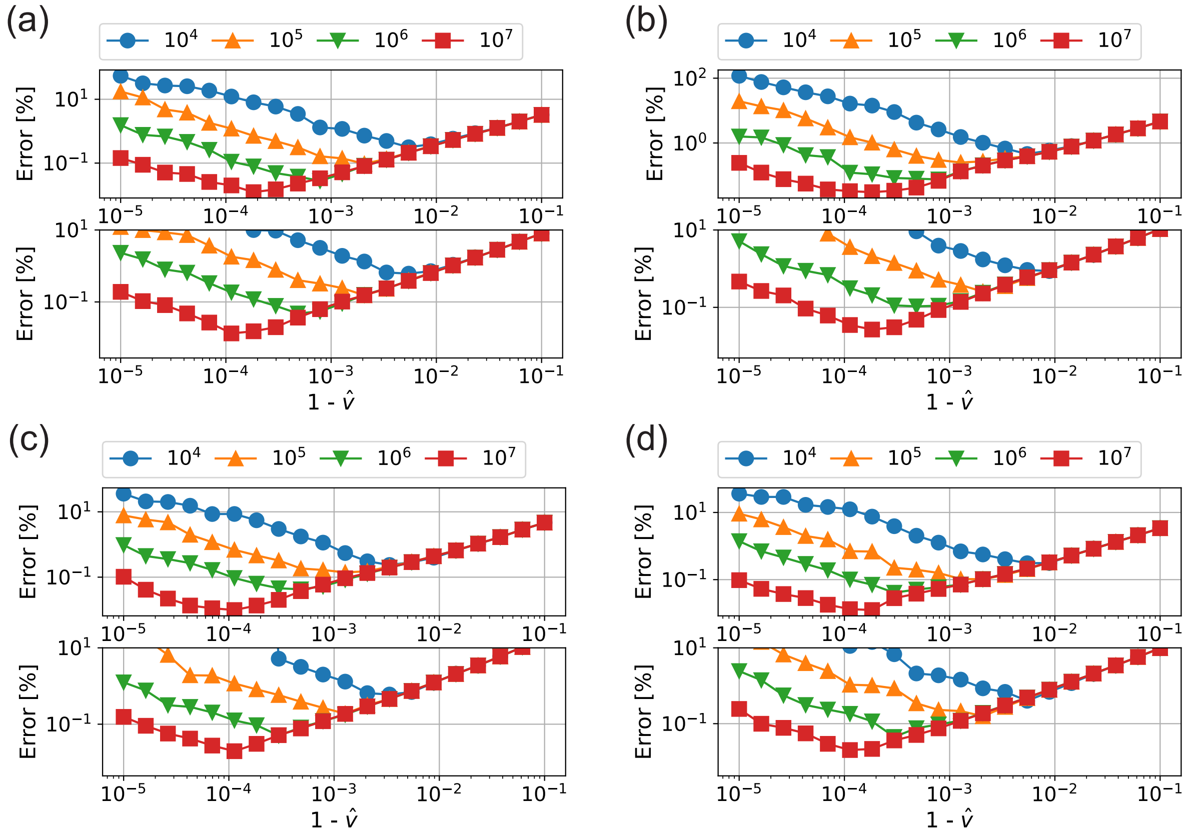

Figure 1: **Numerical conversion analysis.** The gradient errors in the SNNs (5-10-10-3) for various values of $\hat{v}$ and $N_{\text{steps}} \in \{10^4, 10^5, 10^6, 10^7\}$ are plotted. The results are shown for the following neuron models: (a) $\tau_v = \tau_I = \infty$, (b) $\tau_v = \infty$, $\tau_I = 5$, (c) $\tau_v = 2\tau_I = 10$, and (d) $\tau_v = 2\tau_I = 20$. In each subfigure, the upper panel shows the gradient error relating to the first hidden layer and the lower panel shows the gradient error relating to the second hidden layer. The gradient error was calculated by averaging over the 150 data. We set $t^{\text{ref}} = 10$.

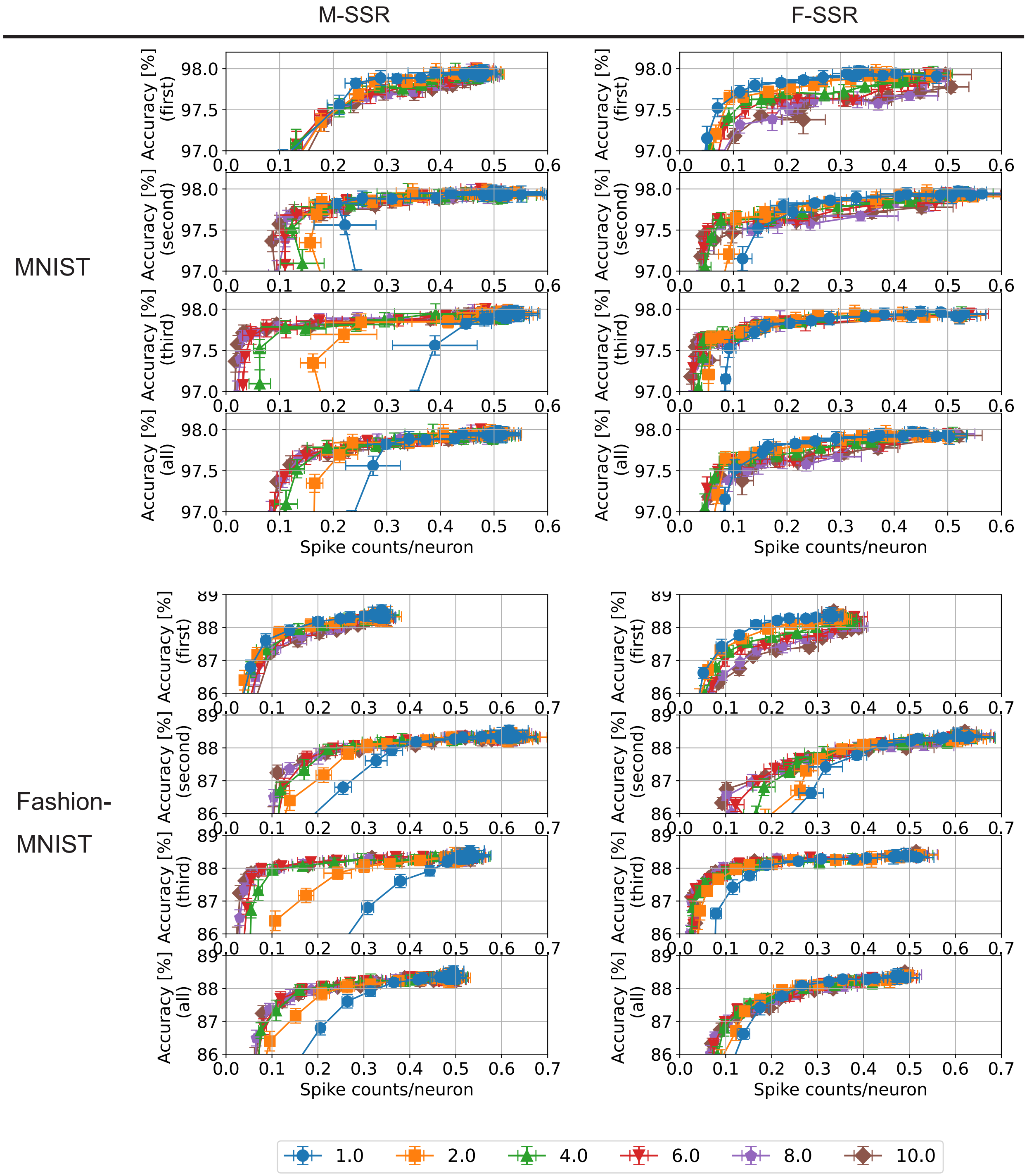

Figure 2: **Effect of ξ on the sparsity–accuracy tradeoff.** The sparsity–accuracy tradeoffs are plotted for SNNs (784-400-400-400-10) with M-SSR and F-SSR for various values of ξ. For each dataset, the first three panels present the sparsity–accuracy tradeoff for the first, second, and third hidden layers, from the top. The bottom panel presents the sparsity–accuracy tradeoff for the sparsity averaged over the three hidden layers. We used the following hyperparameters: $t^{\mathrm{ref}} = 9,\ \gamma_1 = 10^{-4}, \eta = 10^{-4}$, and $\tau_{\mathrm{soft}} = 0.9$.
